# Homoeopathic Management of Hypothyroidism: A Narrative Review of Reported Clinical Experience, Individualised Prescribing, and Research Gaps

**DOI:** 10.7759/cureus.115269

**Published:** 2026-08-26

**Authors:** Pankaj N Lathiya, Pathikkumar Jayeshbhai Patel, Radha Mangukiya, Harshna S Goti, Vaibhaviben Jayeshsinh Parmar, Abhishek P Kulkarni

**Affiliations:** 1 Department of Community Medicine, Research Methodology, Biostatistics, Vidhyadeep University, Surat, IND; 2 Department of Organon of Medicine, Vidhyadeep University, Surat, IND; 3 Department of Pathology and Microbiology, Vidhyadeep University, Surat, IND; 4 Department of Homoeopathic Repertory and Case Taking, Vidhyadeep University, Surat, IND; 5 Department of Practice of Medicine, Vidhyadeep University, Surat, IND; 6 Department of Homoeopathic Materia Medica, Vidhyadeep University, Surat, IND

**Keywords:** autoimmune thyroiditis, constitutional prescribing, homoeopathy, hypothyroidism, thyroidinum

## Abstract

Hypothyroidism is a chronic thyroid disorder associated with fatigue, cold intolerance, weight gain, constipation, menstrual disturbance, hair fall, mood changes, cognitive slowing, and reduced quality of life. This narrative review synthesises published literature on homoeopathic management of hypothyroidism, with emphasis on individualised prescribing, reported symptom and biochemical outcomes, quality of life, and methodological and reporting limitations. The final review incorporated 53 clinical, methodological, and contextual sources. The literature includes observational studies, case series, case reports, and other descriptive sources involving overt hypothyroidism, subclinical hypothyroidism, and autoimmune thyroiditis. Reported approaches include individualised remedies, Thyroidinum and other sarcodes, intercurrent prescriptions, and follow-up-based modification of treatment. Reported outcome domains include thyroid-stimulating hormone, free triiodothyronine, free thyroxine, thyroid antibodies, fatigue, bowel function, menstrual symptoms, weight change, hair fall, mood, sleep, and quality of life. The available evidence is heterogeneous and predominantly uncontrolled, with variation in thyroid subtype, intervention reporting, potency, repetition, concurrent treatment, follow-up duration, and outcome assessment. Reported improvements therefore cannot be assumed to represent treatment-specific effects and may be influenced by natural disease variation, spontaneous thyroid-stimulating hormone fluctuation, regression to the mean, placebo or contextual effects, and concurrent care. A scientifically established mechanism for specific clinical effects of highly or ultra-highly diluted homoeopathic preparations has not been demonstrated, and the broader evidence base does not establish consistent treatment-specific efficacy beyond placebo or contextual effects. Homoeopathic care should not replace or delay indicated thyroid hormone therapy, particularly in overt hypothyroidism, pregnancy, severe thyroid dysfunction, or clinical deterioration. More rigorous controlled studies with clearly defined thyroid subtypes, standardised clinical and biochemical outcomes, documentation of concurrent conventional treatment, and adequate follow-up are required to determine whether any specific therapeutic effect exists.

## Introduction and background

Hypothyroidism is a thyroid disorder characterised by inadequate thyroid hormone production or action and is primarily evaluated using serum thyroid-stimulating hormone (TSH) and free thyroxine concentrations [1-3]. Its clinical presentation ranges from minimal or non-specific symptoms to overt disease associated with fatigue, cold intolerance, weight gain, constipation, dry skin, hair changes, menstrual disturbance, cognitive complaints, and impaired functional well-being. Symptom burden varies considerably among patients and does not necessarily correspond directly to the degree of biochemical abnormality. Some manifestations attributed to hypothyroidism may also occur with coexisting metabolic, nutritional, psychological, or sleep-related disorders; consequently, diagnosis and clinical assessment should be based on appropriate clinical and biochemical evaluation rather than symptoms alone [2,4]. In established primary hypothyroidism, levothyroxine remains the standard thyroid hormone replacement therapy, and complementary or alternative approaches should not delay thyroid function testing, appropriate medical supervision, or indicated thyroid hormone replacement [5,6]. Homoeopathic literature describes an individualised prescribing framework based on the reported totality of symptoms, physical and mental characteristics, and changes observed during follow-up. Reported approaches include individually selected constitutional medicines, organ-directed preparations, sarcodes such as Thyroidinum, intercurrent medicines, and modification of prescriptions according to subsequent clinical assessment [7,8]. These descriptions represent homoeopathic prescribing practices and should not be interpreted as demonstrating thyroid-specific therapeutic effects. A scientifically established mechanism by which highly or ultra-highly diluted homoeopathic preparations produce specific clinical effects has not been demonstrated, and the broader evidence base has not established consistent treatment-specific efficacy beyond placebo and contextual effects [9]. From a patient safety perspective, homoeopathic care should not replace or postpone established diagnostic evaluation or indicated thyroid hormone treatment, particularly in overt hypothyroidism, pregnancy-associated hypothyroidism, severe thyroid dysfunction, or other circumstances in which inadequate thyroid hormone replacement may carry clinically important risks [5,6].

The thyroid-specific clinical literature on homoeopathic management remains limited. Published reports include retrospective and prospective observational investigations, open-label single-arm studies, small case series, and individual case reports [10-12]. These publications describe changes observed during follow-up in thyroid function measurements, thyroid-related symptoms, menstrual complaints, functional status, or patient-reported well-being. The principal clinical reports considered in this review are uncontrolled and therefore cannot reliably distinguish intervention-specific effects from natural disease course, biological variability, regression to the mean, placebo or contextual effects, concurrent treatment, or other time-dependent influences. This interpretive limitation is particularly important in subclinical hypothyroidism, in which mild biochemical abnormalities may fluctuate over time and should not be assumed to represent treatment-associated change solely based on before-and-after observations.

Interpretation is further complicated by heterogeneity in thyroid subtype, study design, sample size, concurrent treatment, follow-up duration, and outcome assessment. Conventional treatment exposure and patient-reported outcomes may themselves influence the interpretation of clinical status and quality of life and therefore require explicit documentation [6,13]. Some reports describe symptomatic change without complete numerical thyroid function data, whereas others provide biochemical observations without sufficient information on diagnostic criteria, concurrent care, attrition, or adverse events. Observational studies, case series, and case reports can generate clinically relevant hypotheses and document treatment chronology, but their uncontrolled designs limit causal inference and prevent reported changes from being regarded as evidence of established clinical efficacy [11,12,14]. Homoeopathy-specific reporting guidance recommends a sufficiently detailed description of clinical history, the nature of the homoeopathic intervention, medicine nomenclature and preparation, dosage, objective outcomes, reported aggravations, and assessment of possible causal attribution [15]. Incomplete reporting of these elements reduces reproducibility and limits interpretation of the available evidence.

A focused narrative review is therefore warranted to organise the reported literature according to thyroid subtype, study design, patient characteristics, prescribing approach, concurrent conventional treatment, biochemical outcomes, patient-reported outcomes, follow-up duration, and methodological limitations. Reported improvements should be interpreted as observations occurring during treatment rather than as established treatment effects unless supported by appropriately controlled evidence. The present review critically evaluates the available literature, distinguishes descriptions of homoeopathic practice from evidence of efficacy, and identifies priorities for more rigorous, transparent, and clinically safe investigation.

Objectives of the review

This narrative review aims to critically synthesise the reported literature on homoeopathy in hypothyroidism, with emphasis on thyroid subtype, study design, individualised prescribing approaches, biochemical and patient-reported outcomes, concurrent conventional treatment, follow-up duration, and methodological limitations. It also aims to identify reporting deficiencies, distinguish uncontrolled clinical observations from established treatment effects, and outline priorities for rigorous future investigation without supporting the replacement or delay of indicated thyroid hormone therapy.

## Review

Methodology

A literature-based narrative review approach was used to examine the homoeopathic management of hypothyroidism. Studies and relevant scholarly sources published between January 2016 and July 2026 were searched through PubMed/MEDLINE, Google Scholar, Scopus, Web of Science, ScienceDirect, the Cochrane Library, and complementary medicine databases. The 2016-2026 publication window was predefined to prioritise contemporary evidence consistent with current diagnostic classifications, thyroid function testing methods, biochemical monitoring practices, and clinical reporting standards. Restricting the period also facilitated greater comparability in interpreting treatment approaches and reported outcomes. The search combined thyroid-related and homoeopathy-related terms using the Boolean operators “AND” and “OR”. The core search string was as follows: (“hypothyroidism” OR “subclinical hypothyroidism” OR “autoimmune thyroiditis” OR “Hashimoto thyroiditis” OR “thyroid dysfunction”) AND (“homoeopathy” OR “homeopathy” OR “homoeopathic medicine” OR “homeopathic medicine” OR “individualised homoeopathy” OR “constitutional prescribing” OR “Thyroidinum”) AND (“thyroid-stimulating hormone” OR “free triiodothyronine” OR “free thyroxine” OR “thyroid antibodies” OR “symptom outcomes” OR “quality of life”).

The wording and field structure of this search were adapted to the syntax of each database. Reference lists of relevant articles were also reviewed to identify additional eligible sources. This article was prepared as a comprehensive narrative review rather than a systematic review or meta-analysis. The Scale for the Assessment of Narrative Review Articles (SANRA) was used as a general reporting framework [16]; Preferred Reporting Items for Systematic Reviews and Meta-Analyses (PRISMA) and Joanna Briggs Institute (JBI) systematic review procedures were not applied. As the literature search was undertaken for narrative synthesis rather than as a formal record-by-record systematic screening process, PRISMA-style counts of records identified, deduplicated, screened, and excluded were not generated. The predefined eligibility criteria and relevance to the objectives of the review guided source selection. Given the narrative design and heterogeneity of the available literature, no formal risk-of-bias or critical appraisal tool was applied. The evidence was instead interpreted according to study design, sample size, comparator use, completeness of intervention and outcome reporting, concurrent treatment, and follow-up duration. The inclusion criteria comprised English-language human studies, clinical reports, observational studies, case series, case reports, and review articles focused on the homoeopathic management of hypothyroidism, subclinical hypothyroidism, autoimmune thyroiditis, or thyroid-related functional complaints. Sources were considered eligible when they described homoeopathic remedy approaches, individualised prescribing, sarcodes, organ-affinity remedies, symptom outcomes, biochemical thyroid outcomes, quality-of-life measures, or follow-up response. Exclusion criteria included non-English articles, studies outside the 2016-2026 period, animal-only studies, editorials without clinical relevance, articles focused only on hyperthyroidism or unrelated endocrine disorders, duplicate publications, purely theoretical discussions without thyroid-specific content, and studies in which homoeopathic intervention could not be separated from herbal, nutritional, yoga-based, or other non-homoeopathic therapies.

For the clinical literature discussed in the review, information was charted using a structured narrative framework comprising author and year, country, study design, sample size, patient population, thyroid subtype, intervention, comparator, outcome measures, follow-up duration, main findings, and principal methodological limitations. Representative clinical reports were organised according to thyroid subtype and study design to facilitate the interpretation of the heterogeneous evidence. Findings relating to subclinical, overt, and autoimmune hypothyroidism were considered separately wherever possible because these conditions differ in their natural history, biochemical characteristics, and clinical management. Reported symptomatic or biochemical changes from uncontrolled studies were interpreted as descriptive clinical observations and not as evidence of treatment efficacy or causal therapeutic effects. Particular attention was given to alternative explanations for observed changes, including spontaneous thyroid function variation, regression to the mean, measurement variability, and concurrent care.

No quantitative synthesis, meta-analysis, meta-regression, pooled effect estimate, heterogeneity analysis, or publication bias assessment was undertaken. The available literature differed substantially in design, patient characteristics, thyroid classification, interventions, comparator use, outcome measures, and follow-up periods. Findings were therefore synthesised qualitatively, with emphasis on the consistency of reported outcomes, methodological limitations, clinical interpretability, thyroid subtype, concurrent treatment, potential spontaneous variation, and the distinction between reported clinical observations and established treatment effects.

Review

Homoeopathic Understanding of Hypothyroidism

Homoeopathic literature describes hypothyroidism as a chronic condition associated with functional slowing, metabolic disturbance, and a broad range of physical and psychological symptoms. Commonly reported features include fatigue, cold intolerance, weight gain, constipation, dry skin, alopecia, menstrual irregularity, depressed mood, cognitive dullness, and reduced initiative [17]. Homoeopathic assessment is reported to consider the presenting symptom pattern, physical generals, mental-emotional features, thermal preference, appetite, sleep, perspiration, bowel habits, menstrual history, family tendency, autoimmune background, and disease progression [18]. Within homoeopathic practice, these characteristics may be used to individualise remedy selection rather than applying a uniform prescription to all patients with similar biochemical findings [19]. Constitutional susceptibility, stress-related factors, emotional patterns, and other individual characteristics have also been discussed as components of homoeopathic case interpretation [20]. These concepts should be understood as features of the homoeopathic interpretive framework and have not been established as causal mechanisms of hypothyroidism through robust biomedical evidence. Miasmatic assessment and detailed case analysis are also described within homoeopathic practice, including in reports concerning patients with chronic or autoimmune thyroid-related complaints [21].

Reported approaches include constitutional prescribing, organ-directed preparations, sarcodes, intercurrent medicines, and modification of prescriptions during follow-up [7,11]. Thyroidinum has been described in the homoeopathic literature as a sarcode used as an adjunctive or intercurrent preparation in hypothyroidism [22,23]. These descriptions represent reported prescribing practices and should not be interpreted as evidence of established thyroid-specific efficacy. Follow-up in published clinical reports may include the assessment of thyroid-related symptoms, functional status, and biochemical thyroid parameters [11,24]. Because the available literature is dominated by uncontrolled observational and case-based evidence, reported changes require cautious interpretation and cannot by themselves establish a causal therapeutic effect. Table 1 summarises representative thyroid-specific clinical reports according to thyroid subtype, study design, intervention, reported outcomes, and principal limitations.

**Table 1:** Clinical evidence in homoeopathic hypothyroidism TSH: thyroid-stimulating hormone Created by the authors based on supporting literature [8,11,12,14,25,26].

Study	Thyroid subtype/population	Study design	Intervention	Outcomes reported	Main findings and limitations
Grelle and Camacho [12]	Subclinical hypothyroidism	Case series of 19 patients	Individualised homoeopathic treatment	Serial TSH measurements and predefined clinical response	Changes in TSH were reported during follow-up. The retrospective uncontrolled design, small sample, and possibility of spontaneous TSH normalisation prevent causal attribution
Ray et al. [8]	Hypothyroidism	Open-label, prospective, single-arm clinical trial	Individualised homoeopathic treatment	Clinical, functional, and thyroid-related outcomes	Improvement was reported during follow-up; however, the absence of randomisation, blinding, and a comparator group substantially limits conclusions regarding efficacy
Arora and Goel [11]	Subclinical hypothyroidism in young women	Prospective observational study	Individualised homoeopathic treatment	Clinical symptoms and thyroid function parameters	Clinical and biochemical changes were reported. The uncontrolled observational design and restricted population limit causal inference and generalisability
Dogra and Mandal [14]	Hypothyroidism	Report of three cases	Homoeopathic treatment	Clinical symptoms and serial thyroid function findings	Symptomatic and biochemical changes were described, but a three-patient uncontrolled series cannot distinguish treatment effects from spontaneous variation, regression to the mean, or concurrent influences
Wadhwa and Singh [25]	Overt hypothyroidism	Single-patient case report	Individualised homoeopathic medicine (Iodium)	Clinical and thyroid function outcomes	The report documents an individual clinical course but cannot establish efficacy or generalisability because there is no comparator and the evidence is limited to one patient
Sabud et al. [26]	Subclinical hypothyroidism	Single-patient case report	Individualised homoeopathic intervention	Symptoms and serial thyroid function values	Clinical and biochemical improvement was described during follow-up; however, a single uncontrolled case cannot exclude spontaneous improvement, regression to the mean, placebo or contextual effects, or concurrent care

Constitutional Assessment and Individualised Case Analysis

Constitutional assessment in the homoeopathic management of hypothyroidism considers physical, mental, emotional, metabolic, and hereditary characteristics in addition to the diagnostic label and thyroid profile [25]. Case analysis may include temperament, thermal preference, appetite, thirst, sleep, perspiration, bowel habits, menstrual history, skin and hair changes, fatigue pattern, mood, stress, and other features used within homoeopathic individualisation [26,27]. Within this framework, remedy selection is based on the overall symptom pattern rather than on thyroid biochemistry alone. Physical generals, mental-emotional characteristics, and associated symptoms may therefore contribute to differentiation among individual prescriptions [28].

Miasmatic assessment is also described in some homoeopathic case analysis frameworks, although its interpretation is subjective and has not been established as a biomedical mechanism of thyroid disease [29]. Family history, thyroid autoimmunity, metabolic comorbidity, menstrual disturbance, and other clinical features remain relevant to the conventional assessment of thyroid disease and its broader clinical context [30]. Follow-up should include appropriate clinical and biochemical measures, including TSH, free thyroid hormones where indicated, relevant thyroid antibodies, symptom burden, functional status, and quality of life [31]. Homoeopathy-specific case reporting should also document intervention details sufficiently clearly to permit the evaluation of the temporal relationship between treatment and reported outcomes [32].

Homoeopathic Remedy Approaches in Thyroid Dysfunction

Homoeopathic prescribing in thyroid dysfunction has been described as an individualised process based on symptom totality, constitutional characteristics, and longitudinal clinical assessment [26]. Reported thyroid-related symptoms include fatigue, cold intolerance, weight change, constipation, menstrual disturbance, hair and skin changes, mood symptoms, and other manifestations that may contribute to the clinical presentation of hypothyroidism [33]. Within homoeopathic literature, remedy differentiation may additionally consider characteristic symptoms, modalities, mental-emotional features, physical generals, family tendency, autoimmune background, and disease progression [19]. Detailed case-taking and individualised prescribing have also been described in case reports involving subclinical and autoimmune thyroid disorders [21,26]. Patients with similar biochemical classifications may nevertheless differ substantially in symptom burden, clinical context, and treatment requirements; TSH values alone do not capture all aspects relevant to clinical management [34]. Within homoeopathic practice, such clinical variability is used as part of the rationale for individualised remedy selection, although this prescribing principle should not be interpreted as evidence of therapeutic efficacy.

**Figure 1:**
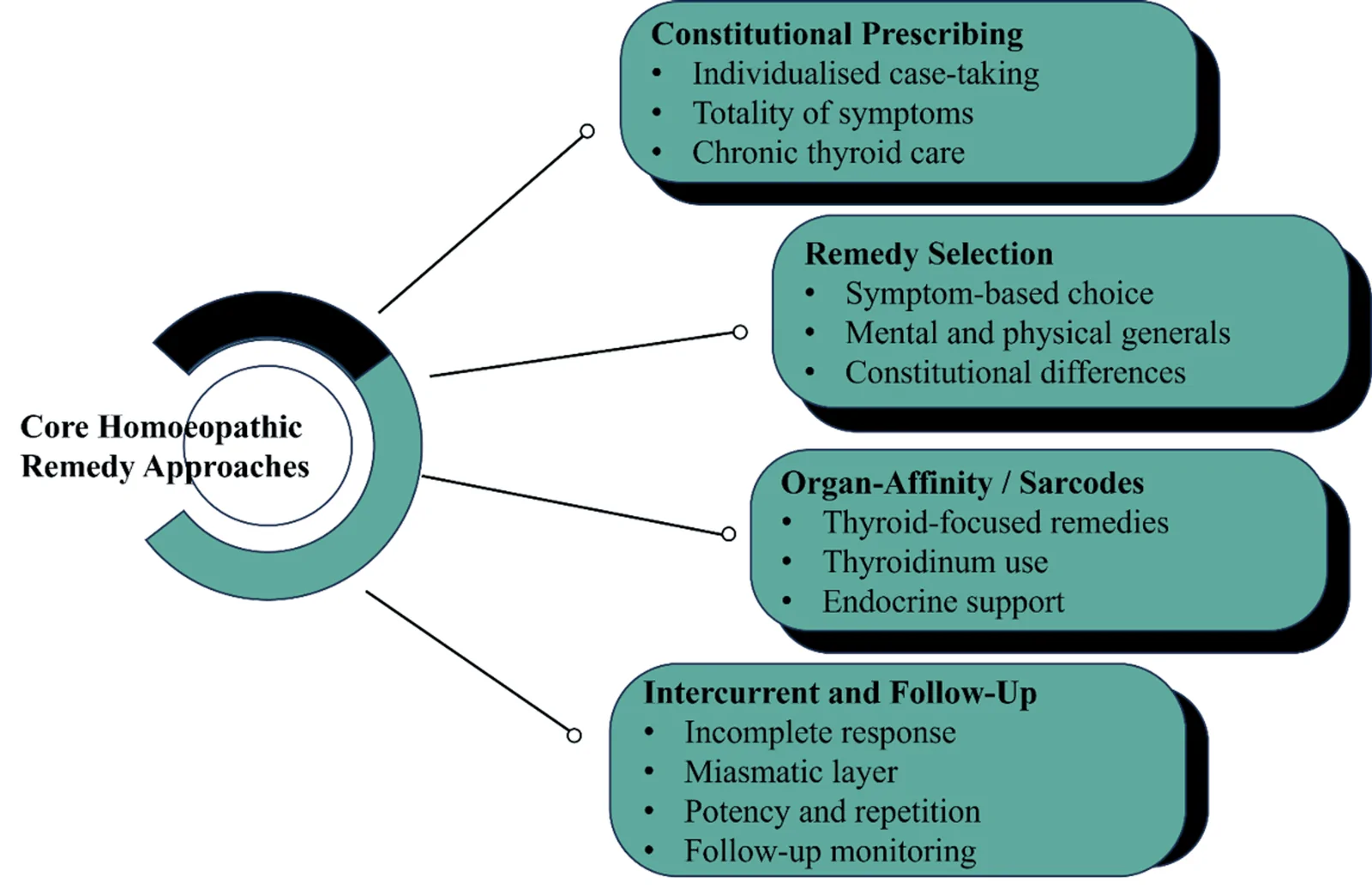
Homoeopathic remedy approaches in thyroid dysfunction Created by the authors using Microsoft PowerPoint (Microsoft Corporation, Redmond, Washington, United States), based on supporting literature [3,4,13,18].

Organ-directed preparations, mother tinctures, and related homoeopathic approaches are described in the broader homoeopathic literature [35]. Thyroidinum has specifically been reported as an adjunctive or intercurrent preparation in hypothyroidism [7]. Its use represents a reported prescribing practice rather than evidence of an established thyroid-specific pharmacological effect. Clinical reports also describe the modification of prescriptions during follow-up according to the observed course of symptoms and thyroid-related findings [8]. Intervention details, including the medicine, potency, dose, repetition, treatment duration, prescription changes, and relevant clinical or biochemical outcomes, should be reported transparently to support reproducibility and critical interpretation [32]. Figure 1 summarises the principal reported homoeopathic prescribing approaches, including constitutional prescribing, individualised remedy selection, organ-directed or sarcode use, and modification of prescriptions during follow-up.

Clinical Evidence on the Homoeopathic Management of Hypothyroidism

Prospective clinical studies, observational investigations, case series, case reports, and practice-based clinical documentation have described the homoeopathic management of hypothyroidism [14,36,37]. Published reports include patients with overt hypothyroidism, subclinical hypothyroidism, autoimmune thyroiditis, and other thyroid-related presentations [11,38]. Commonly reported outcome domains include TSH, free triiodothyronine, free thyroxine, thyroid antibody measurements, fatigue, weight change, constipation, menstrual symptoms, hair loss, cold intolerance, mood-related symptoms, and quality-of-life measures [39,40]. These reports document clinical and biochemical changes observed during follow-up but should not be interpreted as demonstrating therapeutic effectiveness in the absence of appropriate comparator groups. Prospective and observational designs can provide information on the temporal course of symptoms and biochemical measurements during homoeopathic care [41]. Published reports may include thyroid profiles, clinical symptoms, details of the selected intervention, dosage, and follow-up testing [14,40]. Changes in thyroid-related symptoms and biochemical parameters have also been reported in subclinical hypothyroidism and other early or mild thyroid dysfunction [42]. Such findings remain difficult to interpret causally because spontaneous biochemical variation, regression to the mean, placebo and contextual effects, concurrent conventional treatment, and other time-dependent influences cannot be excluded in uncontrolled studies. Case-based evidence may therefore be useful for documenting clinical chronology and generating hypotheses when laboratory values, intervention details, and follow-up are adequately reported, but it cannot establish treatment efficacy.

Heterogeneity is a major limitation of the available evidence. Study design, thyroid subtype, patient selection, outcome domains, intervention reporting, potency, repetition, and follow-up duration vary substantially across publications [36]. This variability limits direct comparison and should be regarded as a methodological limitation rather than evidence supporting the effectiveness of individualised prescribing. Transparent documentation, standardised biochemical assessment, validated symptom and quality-of-life measures, and clear reporting of concurrent treatment are important for improving interpretability [32]. Evidence mapping and larger well-characterised datasets may help identify research gaps and patterns requiring further investigation, but associations observed in uncontrolled datasets should not be interpreted as causal treatment effects [43]. Table 2 shows the principal clinical evidence domains in homoeopathic thyroid care and highlights the methodological limitations that should guide the interpretation of the reported findings.

**Table 2:** Evidence domains and interpretive limitations in homoeopathic hypothyroidism TSH: thyroid-stimulating hormone Created by the authors based on supporting literature [8,10,11,14,21,25,27,26,38].

Evidence domain	What the available literature can contribute	Principal interpretive limitations	Appropriate interpretation	Citation
Prospective or longitudinal clinical reports	Serial assessment of thyroid-related symptoms, thyroid function parameters, intervention details, and follow-up changes	Frequently uncontrolled; limited sample size; potential concurrent treatment; no reliable separation of intervention effects from natural variation	Useful for describing temporal changes during treatment, but not sufficient to establish causality	[8,11]
Retrospective observational evidence	Provides information on real-world prescribing patterns, recorded outcomes, and clinical follow-up in routine practice	Selection bias, incomplete records, uncontrolled confounding, missing data, and variable follow-up	Descriptive and hypothesis-generating; associations should not be interpreted as treatment effects	[10]
Case series	Permits detailed description of individualised prescribing, clinical chronology, laboratory findings, and follow-up	Small samples, no comparator group, selective reporting, regression to the mean, and limited generalisability	Useful for identifying clinical observations and research questions, but unable to establish efficacy	[14]
Individual case reports	Provide detailed patient-level chronology, intervention rationale, biochemical findings, and clinical response	Cannot distinguish a specific treatment effect from spontaneous change, placebo/contextual effects, concurrent care, or measurement variability	Very-low-certainty, hypothesis-generating evidence	[25,27]
Subclinical hypothyroidism evidence	Allows assessment of symptoms and thyroid function trends in a clinically distinct subgroup	TSH may fluctuate or normalise spontaneously; treatment thresholds and natural history differ from overt hypothyroidism	Findings require particularly cautious interpretation and should not be extrapolated directly to overt disease	[26]
Autoimmune or overt hypothyroidism reports	Describe prescribing approaches and reported outcomes in clinically distinct thyroid presentations	Very limited controlled evidence; variation in autoimmune status, disease severity, and conventional treatment	Should be interpreted separately from subclinical hypothyroidism and not pooled conceptually as equivalent evidence	[21,38]

Clinical Documentation and Biochemical Outcome Considerations

Clinical documentation is important when symptoms, constitutional features, and biochemical parameters change at different rates during follow-up [8,26]. Hypothyroidism may involve physical, psychological, metabolic, and reproductive manifestations; therefore, systematic recording is necessary for meaningful clinical assessment [2,33,44]. Relevant clinical information may include presenting complaints, duration of illness, thyroid-related symptoms, menstrual history, bowel habits, weight pattern, sleep, mood, fatigue, hair and skin changes, autoimmune features, metabolic comorbidity, and other factors relevant to the overall clinical picture [28]. Biochemical monitoring provides an objective basis for assessing thyroid status over time. Relevant parameters may include TSH, free triiodothyronine, free thyroxine, thyroid peroxidase antibodies, and thyroglobulin antibodies where clinically indicated [45]. Baseline and follow-up measurements are particularly important because symptoms such as fatigue, weight change, constipation, hair loss, low mood, menstrual irregularity, and cold intolerance are non-specific and may also occur with nutritional, metabolic, psychological, sleep-related, or other chronic conditions [17]. Changes in symptoms should therefore not be assumed to represent biochemical recovery, and changes in thyroid function tests should likewise be interpreted in their clinical context [33].

**Figure 2:**
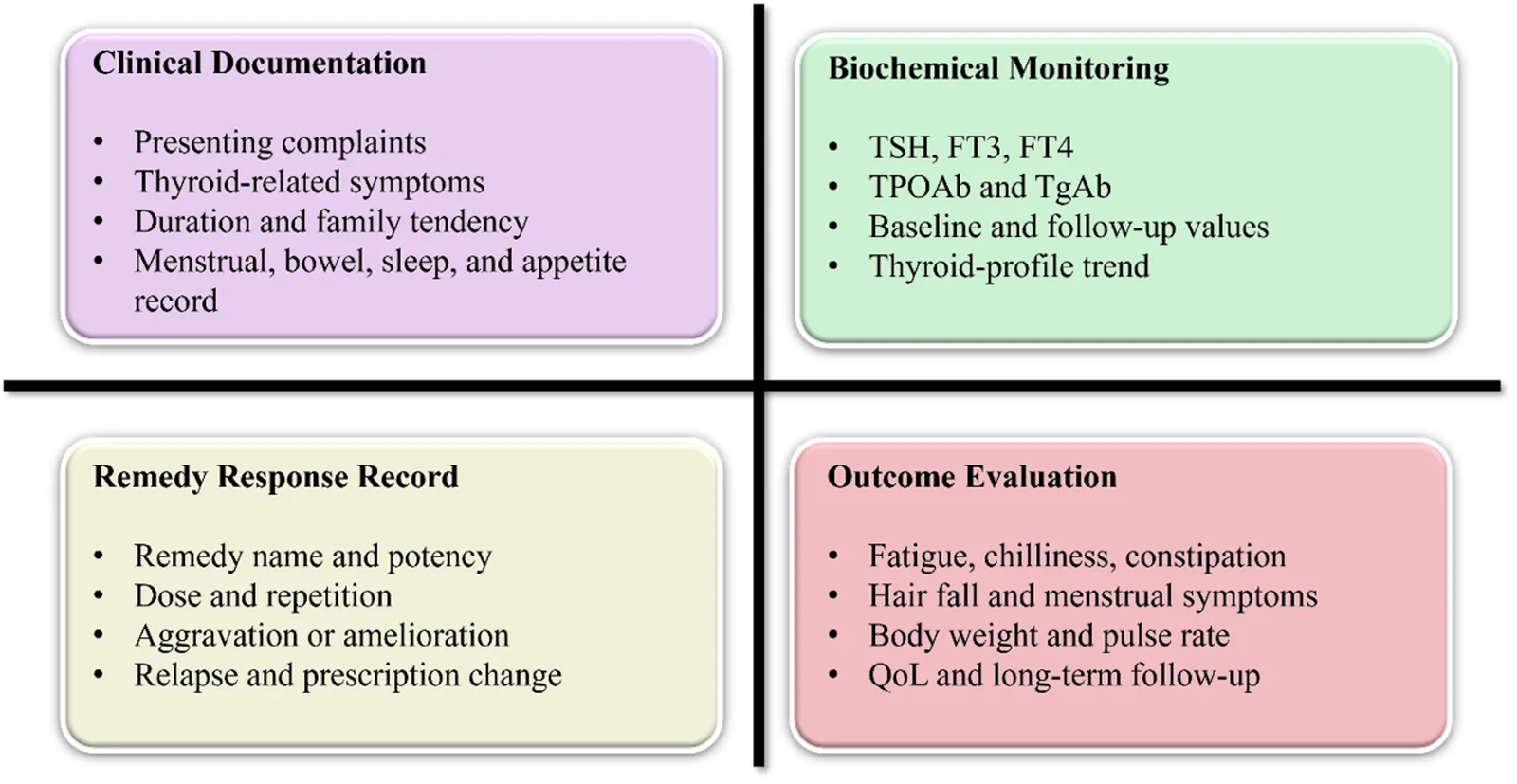
Clinical documentation and outcome assessment in homoeopathic thyroid care TSH: thyroid-stimulating hormone, FT3: free triiodothyronine, FT4: free thyroxine, TPOAb: thyroid peroxidase antibody, TgAb: thyroglobulin antibody, QoL: quality of life Created by the authors using Microsoft PowerPoint (Microsoft Corporation, Redmond, Washington, United States), based on supporting literature [1,4,11,20,27].

Homoeopathic follow-up commonly records changes in general well-being, characteristic symptoms, sleep, bowel pattern, menstrual regularity, mood, hair and skin changes, weight tendency, and tolerance of daily activity [26]. Details of the intervention, including the medicine, potency, dose, repetition, treatment duration, and subsequent prescription changes, should be documented sufficiently clearly to permit the critical interpretation of the reported response [32]. Outcome assessment should incorporate both subjective and objective domains. Subjective domains may include fatigue, cold intolerance, constipation, hair changes, menstrual symptoms, mood, cognition, and quality of life, whereas objective domains may include thyroid function measurements, antibody trends where relevant, body weight, pulse rate, and other clinical findings [12,31,40]. Longitudinal follow-up is required to determine whether observed changes are persistent and clinically meaningful rather than transient. Clear documentation improves reproducibility and permits more reliable interpretation of temporal associations between treatment and reported outcomes; however, in uncontrolled studies, such documentation does not by itself establish therapeutic efficacy [32]. Figure 2 shows the key domains of clinical documentation, biochemical monitoring, remedy-response recording, and outcome evaluation used in homoeopathic thyroid care.

Symptom Relief and Quality-of-Life Outcomes

Symptom and quality-of-life outcomes are important components of follow-up because hypothyroidism can affect energy, gastrointestinal function, menstrual health, mood, cognition, sleep, dermatologic features, and daily functioning [13,31]. These outcomes should be interpreted alongside objective thyroid function measurements rather than considered independently, particularly because subjective improvement does not necessarily indicate biochemical recovery. Structured assessment can reduce the subjectivity of outcome reporting and improve comparability between baseline and follow-up observations [15]. Baseline symptom severity, follow-up interval, patient-reported change, and serial thyroid function findings should therefore be recorded consistently [11,12]. Symptoms such as fatigue, mood disturbance, weight change, impaired concentration, and menstrual complaints are non-specific and may also be influenced by nutritional, metabolic, psychological, sleep-related, or other clinical factors [17,20]. Reported symptom improvement should consequently be interpreted in the context of the patient's clinical findings, thyroid status, and natural course of disease, particularly in subclinical hypothyroidism [42]. Validated symptom and quality-of-life instruments can strengthen outcome assessment by providing reproducible measures of patient-reported health status [31]. In uncontrolled homoeopathic studies, changes in symptoms or quality of life should be described as outcomes observed during follow-up rather than as evidence of a treatment-specific effect. Table 3 shows key symptoms and quality-of-life domains relevant to homoeopathic evaluation in hypothyroid patients.

**Table 3:** Patient-centred outcome domains in hypothyroidism QoL: quality of life Created by the authors based on supporting literature [46-50].

Outcome domain	Common clinical indicators	Follow-up focus	Suggested assessment method	References
Physical vitality and functional status	Fatigue, reduced stamina, impaired daily activity	Change in energy and functional capacity over time	Validated patient-reported outcome measure, activity assessment, clinical interview	[46]
Metabolic and general symptoms	Weight change, cold intolerance, constipation	Persistence or change in clinically recognised hypothyroid symptoms	Body weight, bowel history, structured symptom assessment, thyroid function testing	[47]
Dermatologic features	Hair loss, dry skin, brittle nails	Change in thyroid-associated skin and hair manifestations	Clinical examination, visual documentation, patient report	[48]
Reproductive well-being	Menstrual irregularity, fertility concerns, altered reproductive function	Menstrual and reproductive symptom pattern	Menstrual history, cycle record, appropriate clinical evaluation	[49]
Quality of life	Mood, cognition, sleep, work capacity, social functioning	Overall patient-reported health and functional well-being	Validated thyroid-specific or general quality-of-life instrument	[50]

Homoeopathic Management of Autoimmune and Subclinical Hypothyroidism

Autoimmune thyroiditis and subclinical hypothyroidism should be considered separately because they differ in pathophysiology, biochemical presentation, natural history, and treatment implications [30,47]. Autoimmune thyroiditis may be associated with thyroid peroxidase or thyroglobulin antibodies, progressive thyroid dysfunction, fluctuating TSH concentrations, fatigue, weight change, menstrual disturbance, alopecia, dry skin, mood-related symptoms, and reduced energy [48,50]. Subclinical hypothyroidism is generally characterised by elevated TSH with free thyroxine within the reference range and may be associated with subtle, non-specific, or intermittent symptoms [42]. Its biochemical course may vary over time, and mildly abnormal TSH values may not necessarily indicate persistent thyroid failure. Individualised assessment is a characteristic feature of homoeopathic practice, in which prescribing may take account of the overall symptom pattern and patient-specific characteristics [51,52]. Such individualisation represents a prescribing framework rather than evidence that homoeopathic treatment modifies thyroid autoimmunity or restores thyroid function. Case-based reports have described homoeopathic treatment in hypothyroid patients, but these uncontrolled observations should be interpreted cautiously because they cannot establish comparative effectiveness or causality [14,53].

Subclinical hypothyroidism requires appropriate clinical and biochemical monitoring because symptoms may overlap with nutritional deficiency, anaemia, stress-related fatigue, sleep disturbance, metabolic abnormalities, or reproductive and hormonal factors [42,46]. Follow-up may include the assessment of fatigue, bowel function, cold intolerance, menstrual symptoms, hair changes, sleep, mood, functional status, and quality of life [50]. Serial thyroid function testing is important for determining whether biochemical abnormalities persist or change over time. Baseline and follow-up assessment should document TSH and free thyroid hormone concentrations, relevant antibody status where clinically indicated, symptoms, concurrent conventional treatment, and the timing of follow-up [14,40]. Changes in thyroid antibody concentrations should be interpreted cautiously because antibody status and biochemical thyroid function are not interchangeable measures of clinical improvement [42]. In uncontrolled reports, changes in symptoms, TSH, or antibody concentrations cannot reliably distinguish a specific treatment effect from spontaneous variation, regression to the mean, concurrent treatment, or other influences. Future studies should therefore combine clearly defined thyroid subtypes, structured symptom assessment, serial biochemical monitoring, documentation of concurrent treatment, and adequate follow-up while avoiding claims of efficacy that are not supported by controlled evidence [53].

Evidence Mapping and Reporting Standards in Homoeopathic Thyroid Care

Evidence mapping provides a structured method for organising heterogeneous literature into clinically and methodologically relevant domains [43]. The available homoeopathic thyroid literature includes different study designs and reporting formats, with substantial variation in patient selection, thyroid subtype, intervention characteristics, follow-up duration, and outcome assessment [36]. Organising this evidence by predefined categories can help identify areas in which evidence is concentrated, underreported domains, and important methodological gaps [43]. Patient classification should distinguish overt hypothyroidism, subclinical hypothyroidism, autoimmune thyroiditis, post-surgical hypothyroidism, iodine-related thyroid dysfunction, and inadequately specified or mixed thyroid presentations because these conditions differ in their clinical course and interpretation of thyroid function measurements [3,42]. Baseline reporting should therefore clearly describe thyroid status, symptom burden, duration of illness, relevant autoimmune features, comorbidities, and previous or concurrent treatment so that findings from clinically distinct thyroid populations are not interpreted as interchangeable [3]. Homoeopathic intervention reporting should provide sufficient information to permit the reproduction and critical evaluation of the treatment described. Relevant details include the medicine used, potency, dosage form, repetition schedule, treatment duration, rationale for prescription, subsequent prescription changes, and any concurrent conventional treatment [32,52]. Where constitutional prescribing, organ-directed preparations, sarcodes, or intercurrent medicines are reported, these should be identified explicitly as components of homoeopathic practice rather than established biomedical treatment mechanisms. Published descriptions of Thyroidinum and other individualised approaches illustrate the importance of clearly specifying the intervention actually administered [22,52]. Complete intervention reporting does not establish efficacy but improves transparency and allows readers to evaluate the relationship between the reported treatment and subsequent observations.

Outcome reporting should distinguish patient-reported outcomes from objective clinical and biochemical measures. Relevant patient-reported domains may include fatigue, cold intolerance, constipation, hair and skin changes, mood, cognition, sleep, daily functioning, and quality of life [39]. Objective assessment may include TSH, free thyroxine, and other thyroid function measurements where clinically indicated, thyroid antibody status when relevant, body weight, and other prespecified clinical measures [40]. Combining structured symptom assessment with biochemical follow-up provides a more complete description of the patient's clinical course, but changes in either domain should not be assumed to represent a treatment-specific effect in uncontrolled studies. Follow-up duration, adverse clinical changes, relapse or recurrence, changes in concurrent treatment, prescription modifications, and the durability of reported outcomes should also be documented [32]. Where causal attribution to a homoeopathic intervention is proposed, the basis for that attribution should be stated explicitly rather than inferred solely from temporal association. Validated thyroid-specific quality-of-life instruments may further improve the consistency of patient-reported outcome assessment [31]. Standardised reporting across these domains would improve comparability between studies and strengthen the methodological quality of future research without implying therapeutic efficacy from descriptive or uncontrolled evidence.

Limitations and Future Recommendations

The present review is limited by the heterogeneity of the available homoeopathic literature on hypothyroidism. Differences were observed in publication type, diagnostic classification, thyroid subtype, remedy selection, potency, repetition schedule, follow-up duration, and outcome reporting. The restriction to literature published between 2016 and 2026 may also have excluded earlier homoeopathic studies and clinical reports of potential historical or clinical relevance; consequently, this review should be interpreted as a synthesis of contemporary evidence rather than a complete historical account. A formal risk-of-bias assessment using tools such as ROBINS-I, Cochrane RoB 2, or JBI critical appraisal checklists was not undertaken because this was a comprehensive narrative review rather than a systematic evidence synthesis. This limits the certainty with which the methodological quality of individual studies can be compared and should be considered when interpreting the reported findings. Numerous reports emphasised clinical improvement without consistently presenting biochemical outcomes, validated symptom scales, or longitudinal follow-up data. Variability in constitutional assessment and remedy selection methods further limited comparison across reports. The absence of standardised reporting for TSH, free triiodothyronine, free thyroxine, thyroid antibodies, quality-of-life measures, and relapse patterns also reduced the interpretability and comparability of reported clinical responses.

Future studies should focus on the documentation of homoeopathic thyroid management of the thyroid type and subtype, complete remedy details, potency, repetition, homoeopathic prescription details, and a follow-up schedule, all of which should be well structured. Well-designed observational studies and controlled clinical trials with sufficient sample sizes can help fill the gaps. Biochemical outcomes, symptom severity, quality of life, menstrual and metabolic outcomes, relapse status, and duration of improvement should be reported. There should be large practice-based datasets that can offer them useful information on response patterns and the clinical trends of different remedies.

## Conclusions

This review critically synthesises the reported clinical literature on the homoeopathic management of hypothyroidism, with emphasis on constitutional assessment, individualised prescribing, biochemical monitoring, patient-reported outcomes, and methodological limitations. The available evidence is limited predominantly to uncontrolled observational studies, case series, single-arm investigations, and case reports; consequently, reported symptomatic or biochemical changes should not be interpreted as established treatment effects. Particular caution is required in subclinical hypothyroidism because TSH levels may fluctuate or normalise spontaneously and observed changes may also reflect natural history, regression to the mean, contextual effects, concurrent treatment, or measurement variability. Homoeopathic practice is described in the literature through symptom totality, constitutional characteristics, miasmatic assessment, organ-affinity approaches, sarcodes, potency selection, and modification of prescriptions during follow-up. These approaches represent reported prescribing practices rather than evidence of thyroid-specific efficacy. Future research should distinguish overt, subclinical, autoimmune, and other thyroid subtypes; document concurrent conventional treatment; report remedy details and follow-up consistently; and incorporate serial thyroid function testing, validated symptom measures, quality-of-life instruments, adverse event reporting, and adequate follow-up. Well-designed prospective controlled studies with sufficient sample sizes are required before reliable conclusions regarding effectiveness can be made. Homoeopathic care should not delay or replace indicated thyroid hormone therapy, particularly in overt hypothyroidism, pregnancy, severe thyroid dysfunction, or myxoedema.
